# Primary Metabolism in Avocado Fruit

**DOI:** 10.3389/fpls.2019.00795

**Published:** 2019-06-26

**Authors:** Romina Pedreschi, Virgilio Uarrota, Claudia Fuentealba, Juan E. Alvaro, Patricio Olmedo, Bruno G. Defilippi, Claudio Meneses, Reinaldo Campos-Vargas

**Affiliations:** ^1^ Laboratorio de Fisiología Postcosecha y Bioquímica de Alimentos, Facultad de Ciencias Agronómicas y de los Alimentos, Escuela de Agronomía, Pontificia Universidad Católica de Valparaíso, Valparaiso, Chile; ^2^ Facultad de Ciencias de la Vida, Centro de Biotecnología Vegetal, Universidad Andres Bello, Santiago, Chile; ^3^ Unidad de Postcosecha, Instituto de Investigaciones Agropecuarias, INIA La Platina, Santiago, Chile

**Keywords:** *Persea americana*, mannoheptulose, perseitol, oil, fatty acids, amino acids, cell wall

## Abstract

Avocado (*Persea americana* Mill) is rich in a variety of essential nutrients and phytochemicals; thus, consumption has drastically increased in the last 10 years. Avocado unlike other fruit is characterized by oil accumulation during growth and development and presents a unique carbohydrate pattern. There are few previous and current studies related to primary metabolism. The fruit is also quite unique since it contains large amounts of C_7_ sugars (mannoheptulose and perseitol) acting as transportable and storage sugars and as potential regulators of fruit ripening. These C_7_ sugars play a central role during fruit growth and development, but still confirmation is needed regarding the biosynthetic routes and the physiological function during growth and development of avocado fruit. Relatively recent transcriptome studies on avocado mesocarp during development and ripening have revealed that most of the oil is synthesized during early stages of development and that oil synthesis is halted when the fruit is harvested (pre-climacteric stage). Most of the oil is accumulated in the form of triacylglycerol (TAG) representing 60–70% in dry basis of the mesocarp tissue. During early stages of fruit development, high expression of transcripts related to fatty acid and TAG biosynthesis has been reported and downregulation of same genes in more advanced stages but without cessation of the process until harvest. The increased expression of fatty acid key genes and regulators such as *PaWRI1*, *PaACP4-2,* and *PapPK-β-1* has also been reported to be consistent with the total fatty acid increase and fatty acid composition during avocado fruit development. During postharvest, there is minimal change in the fatty acid composition of the fruit. Almost inexistent information regarding the role of organic acid and amino acid metabolism during growth, development, and ripening of avocado is available. Cell wall metabolism understanding in avocado, even though crucial in terms of fruit quality, still presents severe gaps regarding the interactions between cell wall remodeling, fruit development, and postharvest modifications.

## Introduction

Avocado (*Persea americana* Mill) is a rich oil fruit of high economic importance in the international trade. Its nutritional value has been lately highlighted: it is a rich source of monounsaturated and polyunsaturated fatty acids associated with a decreased risk of cardiovascular diseases (Mendez and Hernandez, 2007; Rodriguez-Sanchez et al., 2015). It is also an important source of vitamins A, B, and C and minerals such as potassium, phosphorus, magnesium, iron, and a rich source of fiber and antioxidants (Villa-Rodriguez et al., 2011; Dreher and Davenport, 2013; Bill et al., 2014).

*Persea americana* Mill is quite unique not only from a compositional point of view but also shows a very peculiar and complex physiology compared to other fruit. For example, flowering period can last up to 3 months; thus, a broad range of fruit physiological ages can be on the same tree, which will be evident during postharvest storage and management (Lewis, 1978). Fruit set in addition is extremely low (less than 0.1%). Fruit can hang on the tree for more than 12 months, time far beyond needed to reach physiological maturity to be able to ripen when detached (Hernández et al., 2016).

What turns avocados unique compared to other fruit is the presence of C_7_ sugars (e.g., mannoheptulose and perseitol) instead of C_6_ sugars as main phloem transported sugars and as respiratory substrates (Liu et al., 1999b, 2002; Bertling and Bower, 2005). These C_7_ sugars have been reported as the “tree factor” that inhibits the ripening process of the fruit on the tree (Liu et al., 1999b, 2002; Bertling and Bower, 2005; Landahl et al., 2009; Blakey et al., 2010) and possibly associated with the differences in ripening speed of fruit postharvest (Landahl et al., 2009).

Primary metabolism in avocado, even though an essential component of growth and major component of fruit quality, has been mainly focused on carbon and oil metabolism and to a much lesser extend to amino acids and organic acids. In the decades of the 1960s on, several studies on the role of C_7_ sugars and starch during growth and development of avocado were reported (Bean et al., 1962; Liu et al., 1999a,b; Richings et al., 2000; Cowan, 2004, 2017; Tesfay et al., 2010). Unlike other fruit, avocado accumulates oil instead of sugars, thus oil metabolism during growth, development, and ripening has been of high interest (Salas et al., 2000; Ozdemir et al., 2004; Blakey et al., 2012; Ibarra-Laclette et al., 2015). With the advance in post-genomics tools (e.g., transcriptomics, proteomics, and metabolomics) more integrative studies related to carbohydrate and oil fruit metabolism during growth, development and ripening in avocado have been reported (Hurtado-Fernández et al., 2011, 2015a; Pedreschi et al., 2014; Ibarra-Laclette et al., 2015; Kilaru et al., 2015; Fuentealba et al., 2017; Rodríguez-López et al., 2017) but almost inexistent literature related to organic acid and amino acid metabolism.

Primary metabolism in avocado and other oily fruit undergoes large changes during growth, development, and ripening; thus, the reaction networks or metabolic pathways involved are quite similar, still the set of regulations that directly affect the metabolic fluxes to different destinations remains largely unknown (Beauvoit et al., 2018). Thus, in this review, we aim to: (1) delve into the importance of primary metabolism on quality impact for avocado and (2) provide a deep review of avocado fruit primary metabolism (cell wall, carbohydrates, oil, amino acids, and organic acids) with a main emphasis on studies on carbohydrate and oil metabolism.

### Avocado Origin, Botany, and Commercial Importance

Avocado (*Persea americana* Mill) belongs to the Lauraceae family composed of 50 genera and approximately 2,500–3,000 species (Rohwer, 1993; Chanderbali et al., 2008). Three well recognized horticultural races exist: (1) the Mexican race (*P. americana* var. *drymifolia*) adapted to the tropical highlands; (2) the Guatemalan race (*P. americana* var. guatemalensis, L.O. Williams) adapted preferentially to medium elevations in the tropics; and (3) the West-Indian race (*P. americana* var. *americana*) cultivated in the lowland humid tropics (Chanderbali et al., 2008; Ibarra-Laclette et al., 2015).

Avocado (*Persea americana* Mill) is cultivated throughout the tropical and subtropical regions of the world. A total of 71.9% of the world production come from Americas and Mexico is currently by far the top producer in the world accounting for about 28% of the world production (FAOSTAT, 2018). Commercial avocado production is based on grafting cultivars onto rootstocks mainly of the Mexican and Guatemalan races. In subtropical climates, the Guatemalan genotypes represent the dominant horticultural race (Ibarra-Laclette et al., 2015). *Persea americana* Mill cv. Hass is the most important avocado cultivar worldwide representing 95% of the total volume commercialized. Its international trade has doubled in the span of 6 years (FruitTrop, 2015).

### Avocado Fruit Development: Special Features of *Persea americana*

Avocado fruit follows a single sigmoid curve. Independent of the cultivar (early or late maturing cultivars), the early period of growth is characterized by a rapid cell division (Barmore, 1977). Cell division in the fruit mesocarp goes beyond the initial period of growth but continues during fruit development and continues even in the mature fruit attached to the tree (Van den Dool and Wolstenholme, 1983; Bower and Cutting, 1988). Avocado is a climacteric fruit characterized by an increase in the respiration rate and ethylene production at the onset of ripening. Compared with other climacteric fruits, avocado produces high amounts of ethylene (80–100 μl kg^−1^ h^−1^ at 20°C) at the climacteric peak (Seymour and Tucker, 1993). Fruit does not ripen on the tree, which has been previously attributed to a possible role of C_7_ sugars (mannoheptulose and perseitol) acting as inhibitors and controlling the ripening process (Liu et al., 1999b, 2002; Landahl et al., 2009) and to the low levels of ACC (1-aminocyclopropane-1-carboxylic acid) of fruit attached to the tree (Sitrit et al., 1986), but other studies indicate that additional metabolites might be involved (Pedreschi et al., 2014; Fuentealba et al., 2017).

Avocado fruit development period is quite extensive, between 6 and >12 months from flowering to maturity (Scora et al., 2002). Unlike other fruits that accumulate carbohydrates during growth and development, avocado accumulates mostly oil. Avocado mesocarp fruit starts oil accumulation a few weeks after the fruit sets and oil accumulation continues during growth and development until maturation (Ozdemir et al., 2004). Based on dry weight, approximately, the mesocarp accumulates 60–70% oil and 10% carbohydrates. Oil is stored in the form of triacylglycerol (TAG) and mainly composed of oleic acid (Ozdemir et al., 2004); however, the fatty acid profile of the oil can be largely affected by environmental and geographical conditions (Donetti and Terry, 2014; Pedreschi et al., 2016). Sixty percent of the total carbohydrates, as previously mentioned, is composed of C_7_ sugars (mannoheptulose and perseitol) (Liu et al., 2002).

### General Overview on Primary Metabolism: Primary Pathways and Their Relationship With Fruit Quality

The most integrative studies on primary metabolism comprising information at different levels of cellular control (e.g., genes, proteins and metabolites) orchestrated by hormones have been carried out with tomato as considered as “model fruit.” Major carbon source for fruit is usually sucrose, which is imported from the leaves to the phloem; however, avocado is unusual in which high contents of C_7_ sugars (mannoheptulose and perseitol) are present. Fruit growth and ripening are based on central carbon metabolism, which provides the energy and biosynthetic precursors. These pathways involve sucrose, starch, major organic acids, respiration (Beauvoit et al., 2018), and fatty acid metabolism for a rich fat fruit such as avocado. Unlike other fruit in which the balance of sugars and organic acids is determinant of fruit quality, for avocado, the content of oil and profile of fatty acids is determinant of fruit quality.

Amino acid metabolism is important not only for providing the building blocks for protein synthesis but also for the synthesis of other metabolites (Gonda et al., 2010). Even though amino acids and derivatives can influence fruit quality and taste, very few studies have been carried out with avocado fruit regarding the behavior of different amino acids during fruit growth, development, and ripening.

Firmness is a very important quality parameter of avocado and as such is highly influenced by the composition and architecture of the cell wall. During growth, the cell wall protects and shapes the fruit, and then during ripening its disassembly has strong implications in quality perception and shelf-life. It has been reported that partial cell wall degradation at the ripe stage can provide sugars (e.g., uronic acids), which are phosphorylated and recycled *via* the UDP-sugar pyrophosphorylase, thus providing building blocks for other biosynthetic processes (e.g., protein synthesis, sugar accumulation) (Geserick and Tenhaken, 2013; Beauvoit et al., 2018).

Hormones are crucial during fruit growth and development. Cytokinins and gibberellins are involved in early events after pollination. Fruit growth and development are controlled by coordination of different plant hormones. Up to date, except for a recent study on tomato (Li et al., 2019), information is very limited related to the link between hormonal signals and primary metabolite changes during fruit development and ripening. Auxins and gibberellins promote cell division and expansion, thus influence fruit size. At fruit set, auxins and gibberellins impact the genes involved in primary metabolism (Ruiu et al., 2015). Ethylene and abscisic acid become important for fruit maturation since they tightly control fruit ripening in climacteric fruit. The role of these hormones in avocado ripening has been extensively reported.

## Carbohydrate Metabolism During Avocado Fruit Development and Ripening

Monosaccharides play a key role in primary metabolism by linking energy derivation by photosynthesis to the demands of anabolic and catabolic processes. Monosaccharides are the substrates for sucrose biosynthesis, the major transportable sugar in most vascular plants (Cowan, 2017). However, avocado is quite unique, because C_7_ sugars (mannoheptulose and perseitol) have been reported as the predominant transportable and storage sugars in leaves and fruit of avocado. Still, despite the importance of these C_7_ sugars, there is only sparse information about the synthesis, metabolism, transport, and physiology of C_7_ sugars not only in avocado but also in other species where they occur (Cowan, 2004, 2017). Roles of mannoheptulose and other C_7_ sugars in the regulation of carbon flux and protection against oxidative damage are based on published studies (Cowan, 2004, 2017).

### C_7_ Sugars Occurrence, Biosynthesis, Transport and Compartmentalization in Avocado

Mannoheptulose from avocado fruit was first isolated and identified by LaForge (1916). Mannoheptulose and perseitol have been reported as the major soluble sugars in the phloem sap, leaf petiole exudates, seed, and mesocarp of avocado (Cowan, 2004). Based on perseitol concentrations of 2.5-fold higher in the sap from the trunk of the avocado tree than the sap from the petioles, different sites of synthesis for D-mannoheptulose and perseitol are assumed (Cowan, 2004). A possible route for D-mannoheptulose biosynthesis in avocado fruit involving D-arabinose-5-phosphate ↔ D-ribose-5-phosphate being both substrates converted to hexose 6-phosphate and triose phosphates *via* intermediates that include D-mannoheptulose 7-phosphate and D-altro-heptulose 1,7-bisphosphate has been postulated by Cowan (2004). Similarly, to the pathway of sedoheptulose biosynthesis, D-arabinose-5-phosphate and D-ribose-5-phosphate are then the substrates for transketolase activity and formation of D-manno-heptulose-7-phosphate and 1,7 biphosphate. Arabinose has been reported in substantial amounts in avocado (Jeong and Huber, 2004). By phosphate activity then, D-mannoheptulose would be formed. Cowan (2004) sustains that D-mannoheptulose is a potent inhibitor of respiration and prevents entry of glucose into glycolysis acting as a competitive inhibitor of hexokinase, thus altering the fate of sugar metabolism. Thus, to sustain fruit growth and development, it becomes crucial the effects described above of D-mannoheptulose in order to continue sugar export and phloem loading.

In avocado leaves, Liu et al. (1999a, 2002) reported D-mannoheptulose and perseitol as products of CO_2_ assimilation. D-mannoheptulose is formed in leaves as a phosphorylated product of sedoheptulose 1,7 biphosphate *via* condensation of dihydroxyacetone phosphate and erythrose-4-phosphate (Liu et al., 1999a, 2002). Previously, Häfliger et al. (1999) mentioned that C_7_ sugars can be formed by three well-known enzymatic reactions that form C_7_ intermediates: (1) erythrose-4-P + dihydroxyacetone-P sedoheptulose 1,7-bis P corresponding to an aldose reaction; (2) xylulose-5-P – ribose-5-P sedoheptulose-7-P-glyceraldehyde-3-P corresponding to a transketolase reaction; and (3) a transaldolase reaction – fructose-6-P + erythrose-4-P sedoheptulose-7-P + glyceraldehyde 3-P. All three pathways require NADPH/NADP^+^. Following, Tesfay et al. (2012) reported conversion of D-mannoheptulose to perseitol and vice versa in dry seed, cotyledons, and mesocarp of avocado together with detection of aldolase enzymes possibly a transaldolase present in different avocado tissue types and potentially involved in the conversion of the C_7_ sugar storage form (perseitol) into the C_7_ sugar transport form D-mannoheptulose. But still, as reported by Cowan (2017), the origin of sedoheptulose 7-P and mannoheptulose 7-P as precursors of heptoses and heptitols in plants remains to be elucidated. Both sedoheptulose and mannoheptulose together with sucrose are products of the reductive pentose phosphate pathway in mature source leaves being C_7_ sugar formation stimulated during photosynthesis having as evidence that mature leaves of avocado incorporated CO_2_ into mannoheptulose-7-P, mannoheptulose, sedoheptulose 7-P, sucrose, and perseitol (Cowan, 2017).

Regarding C_7_ sugars transport in avocado trees, previous studies carried out with sap collected from the petioles of mature leaves and sap collected from girdled branches (Liu et al., 1999a,b, 2002; Tesfay et al., 2012) appear to indicate that mannoheptulose and perseitol are the most important and major C_7_ mobile sugars in the tree and readily available for growth and development. Different studies suggest that mannoheptulose is a precursor of perseitol being perseitol conversion independent of photosynthesis (Cowan, 2004, 2017). From studies not only on avocado but also on other C_7_ containing species, Cowan (2017) proposed a C_7_ biosynthesis pathway. C_7_ sugars seem to arrive from CO_2_ in photosynthetic organisms, and there is evidence supporting that sedoheptulose 7-P and mannoheptulose 7-P are precursors of sedoheptulose and mannoheptulose; however, the origin of the phosphorylated precursors is not known. The potential routes include transaldolase catalyzed condensation of dihydroxyacetone from D-fructose-6-P with the aldose D-erythrose-4-P to form sedoheptulose 7-P and then a transketolase catalyzed transfer of an α-hydroxy-acetyl from D-fructose-6-P to the aldopentose D-arabinose-5-P for mannoheptulose 7-P formation (Cowan, 2017). Thus, the proposed hypothesis states that C_7_ sugars originate from fructose-6-P generated from hydrolysis of sucrose *via* a transketolase-dependent-heptulose shunt. Relatively recently, Kilaru et al. (2015) reported a complete transcriptome analysis of developing avocado fruit and found complete glycolytic pathways in cytosol and plastids being sucrose synthase and invertases highly expressed at different fruit developmental stages indicating that sucrose synthase besides being high abundant in the cytosol might be the major actor in the generation of hexoses necessary later for pyruvate synthesis. These authors also reported high expression of orthologs of transketolases and transaldolases in fruit mesocarp plastids suggesting also a possibility for their synthesis in the mesocarp. So possibly, sucrose synthase and invertases provide the supply of fructose for C_7_ sugar biosynthesis (Cowan, 2017).

### Soluble Sugar Metabolism During Avocado Growth, Development, and Ripening

During early stages of growth and development, more than 40% of the mesocarp weight is composed of sugars. Sugars continue to increase and accompany the rapid fruit growth period and then start to decline concomitant with oil accumulation (Kilaru et al., 2015). Liu et al. (2002) reported high total sugar concentrations (up to 44%, 40 and 22% of total dry weight) in the flesh, seed, and peel of *Persea americana* cv. Hass after 2 months of fruit set. Five different soluble sugars were followed during growth and development: sucrose, fructose, glucose, mannoheptulose, and perseitol. These five sugars constituted 98% of the total soluble sugar content of the fruit (Liu et al., 2002). In general, sucrose levels were low in all tissues (seed, peel, and flesh) and did not fluctuate much. For very young fruitlets the predominant sugars in seeds, peel and flesh of avocado corresponded to glucose, fructose, D-mannoheptulose, and perseitol but as fruit rapid expansion phase culminated then decreased levels of fructose and glucose were reported (Liu et al., 2002). In general, the most abundant sugars in avocado corresponded to C_7_ sugars (mannoheptulose and perseitol). During early stages of growth, mannoheptulose corresponded to 12, 19, and 12% of the total soluble sugars in the peel, flesh, and seed, respectively, and perseitol corresponded to 9, 15, and 10% of the total soluble sugars in the peel, flesh and seed, respectively (Liu et al., 2002). Once, surpassed the rapid expansion phase, then mannoheptulose concentrations decreased in seed and flesh but still remained high in the peel (Liu et al., 2002). However, Cowan (2004) reported higher contents of mannoheptulose in avocado cv. Hass mesocarp from 2 months after fruit set on (210 days after fruit set, 78 mg g^−1^) and the contents of perseitol remained stable during early stages of growth and during development (around 55 mg g^−1^). Perseitol instead decreased in the peel and flesh. Legal minimum maturity for Hass avocado in California corresponds to 20.8% (dry matter content) and as fruit matured, D-mannoheptulose decreased but perseitol varied very little. For starch, the peel and flesh of avocado accumulated very little content of starch and did not vary during early stages of development. Instead, for early stages of development the seed accumulated low amounts of starch (<1%) but as it started to increase its weight, starch content in the seed corresponded to 30% of its dry weight (Liu et al., 2002). Cessation of sugar accumulation coincides with oil accumulation. Kilaru et al. (2015) state that the carbon available for oil biosynthesis in avocado fruit seems atypical. High expression of different genes associated with glycolysis as potential source of pyruvate for fatty acid synthesis was reported by Kilaru et al. (2015).

C_7_ sugars have been postulated as the ripening inhibitors of *Persea americana* fruit, while attached to the tree (Liu et al., 1999a,b, 2002) based on findings that C_7_ sugars are phloem mobile, second mannoheptulose is a very potent inhibitor of respiratory processes performing as an hexokinase inhibitor, thus impeding the input of glucose into glycolysis (Board et al., 1995; Liu et al., 2002) and finally based on the inhibition of the ripening process of the fruit until C_7_ sugars are metabolized to below a certain level in the fruit (Liu et al., 2002; Landahl et al., 2009). C_7_ sugars (mannoheptulose and perseitol) may control/trigger the ripening process (Landahl et al., 2009; Meyer and Terry, 2010; Blakey et al., 2012), but other studies indicate that additional players are involved (Pedreschi et al., 2014; Fuentealba et al., 2017). Fuentealba et al. (2017) reported higher expression of a chloroplastic transketolase enzyme in the mesocarp tissue of slow ripening Hass avocados. Previous works have reported that C_7_ sugars together with sucrose and C_6_ sugars are used during the ripening process (Liu et al., 1999b; Landahl et al., 2009). Liu et al. (2002) reported for mature but unripened fruit, perseitol as the main form of carbohydrate (~30 mg g^−1^ dry weight) and greatly exceeded the levels of sucrose, fructose, and glucose and starch, while mannoheptulose levels remained low during the ripening process. This ripening process was accompanied by increases in C_6_ sugars, while contents of perseitol and starch substantially decreased (Liu et al., 2002). In fully ripe fruit, perseitol declined to levels below those of sucrose and hexoses (Liu et al., 2002). Similarly, after harvest and during ripening of avocado cv. Hass allowed to ripen at room temperature, Blakey et al. (2012) reported a decline in the mesocarp content of mannoheptulose and perseitol in fruit from three different locations and increases and decreases for glucose, fructose and sucrose during ripening for the fruit from these three different locations. The increase of free glucose during avocado fruit ripening was associated to the high amounts of cellulose produced by avocado fruit during ripening possibly stimulating the synthesis of abscisic acid and ripening. The low correlations found between C_7_ sugar concentrations and respiration or total protein content were attributed to the fact that C_7_ sugars are used in several cellular processes (Blakey et al., 2012).

Avocado cv. Hass dominates the international trade with more than 95% participation. Depending on the origin country and distance to the market, avocados are transported at low temperature and with controlled atmosphere conditions. Under these transport or storage conditions, the content and role of C_7_ sugars and total soluble sugars of the fruit are key to reach such markets with excellent fruit quality. These C_7_ sugars have also been reported to act as antioxidants in the mesocarp of avocado and thus its initial content at harvest correlated to the storability of the fruit conferring not only carbohydrates to sustain respiration but also stress-protection agents (Tesfay et al., 2010).

## Metabolism of Organic Acids During Avocado Fruit Growth, Development, and Ripening

Organic acids in fruit can act as intermediate in diverse metabolic pathways, as precursors for the synthesis of amino acids, plant hormones, fatty acids, secondary metabolites, and cell wall components (Walker and Famiani, 2018). The most known organic acids malic, citric, isocitric, galacturonic, oxalic, and tartaric are quite abundant in some fruit, and the phenolic acids and ascorbic acid are present in all fruit. In fruit in general, most of the malate and citrate of the pulp is localized in the vacuole, and most of the organic acids present in the pulp are not imported but synthesized in the pulp from transported sugars (Etienne et al., 2013; Famiani et al., 2015).

Not much work up to our knowledge has been reported regarding the most known organic acids evolution during growth, development, and ripening of *Persea americana* possibly due to its high oil content that significantly contributes to fruit quality and taste instead of other fruit in which organic acids such as malate, citrate, tartaric, etc. play a significant role in fruit quality and taste. Avocado is classified as a non-acid fruit and compared to other fruit, and it contains very low amounts of citric and malic acid being the tartaric acid the predominant organic acid (Duckworth, 1966; Ahmed et al., 2010; Viña et al., 2013; Defilippi et al., 2015). Defilippi et al. (2015) reported the organic acid profile of Hass avocado at harvest and during ripening at 20°C up to 15 days. Total acids (sum of tartaric, malic, citric and ascorbic) tended to decrease as ripening advanced and correlated to the decrease of titrable acidity during this period. This observed decrease was mainly governed by the drastic decrease of malic acid. Tartaric acid remained constant during this period and the contribution of ascorbic acid and citric acid was low and remained relatively constant during this period (Defilippi et al., 2015). Ahmed et al. (2010) reported for avocado cv. Fuerte, a mild decrease of titrable acidity during ripening as well as a mild but significant decrease of ascorbic acid during this period. In addition, Arias et al. (2012) reported higher acidity of the seed than the peel and mesocarp in avocado cv. Hass from Algarve, but the acidity of the mesocarp was higher (0.04% citric acid) than the value reported for avocados cv. Hass from Mexico. At harvest, Fuentealba et al. (2017) reported at the protein level differences in expression of enzymes involved in the metabolism of organic acids such ATP dependent citrate synthase, cytosolic NAD malate dehydrogenase and for Hass avocado mesocarp displaying differences in ripening speed suggesting that the speed of ripening is dependent on primary metabolism. Citrate, isocitrate, and malate are intermediates of the Krebs cycle, thus changes in their content during growth, development and ripening are expected. Most of the Krebs cycle acids are stored in the vacuole (Etienne et al., 2013). During avocado ripening organic acids could be used as substrates in the glycolytic pathway, trimesic acid cycle, and gluconeogenesis (Mu et al., 2018); thus, the slight but significant decreases of total acids in avocado during ripening could be expected.

As stated by Mu et al. (2018), genetic regulation of organic acids not only is complex but involves several polygenes, and the fruit organic acid content is correlated with the activities of related enzymes. It has been reported for example that phosphoenolpyruvate carboxylase (PEPC), NAD-dependent malate dehydrogenase (NADMDH), and NADP-malic enzyme (NADP-ME) are important for the synthesis of malic acid and citric acid, but NADP-ME plays a major role in the degradation of malic acid (Mu et al., 2018). Related to *Persea americana*, Ibarra-Laclette et al. (2015) reported the transcriptome of the Mexican avocado and different organs (seeds, roots, stems, leaves, aerial buds, and flowers) and for three different fruit ripening stages defined as pre-climacteric, climacteric, and post-climacteric. A higher number of genes related to organic acid metabolic processes at the pre-climacteric stage followed by the climacteric and post-climacteric stages were reported by Ibarra-Laclette et al. (2015). Annotated reported polygenes corresponded to malate dehydrogenase, peroxisomal NAD malate dehydrogenase, malate synthase, aluminum activated malate transporter 9, aconitase, phosphoenolpyruvate carboxylase, and phosphoenol pyruvate carboxylase kinase, but their expression patterns at the three different stages previously mentioned are not available (Ibarra-Laclette et al., 2015). Kilaru et al. (2015) reported as supplementary information the pattern expression of key genes related to organic acid metabolism in Hass avocado at five different developmental stages (from 75 g fruit and 4% oil to 200 g fruit and 12% oil). The gene annotated as phosphoenolpyruvate carboxylase family protein increased in expression from stage I and II but then tended to decrease in expression reaching its lowest level at stage V corresponding to mature fruit. Genes annotated as malate dehydrogenase cytosolic and plastidial did not present clear differences in expression at the five different developmental stages. As previously mentioned, avocado is considered as non-acid fruit and previous studies concluded that there is no correlation between the expression of organic acid metabolism related genes and organic acid content (Mu et al., 2018).

Regarding the second metabolic group of organic acids represented by ascorbic and tartaric acid, information regarding the evolution of ascorbic acid in Hass avocado mesocarp, exocarp, and seed is available. Ascorbic acid is located in all compartments of the cell. Tesfay et al. (2010) reported ascorbic acid contents for Hass avocado fruit from 16 weeks after full bloom (~70 g fresh weight) to commercial maturity (~180 g fresh weight, 18–22% oil content FW) lowest in the mesocarp and highest in the exocarp. During these 6 months of growth and development, avocado fruit displayed average contents of ascorbic acid of 0.41 mg g DW^−1^ of mesocarp tissue, 2.77 mg ascorbic acid g DW^−1^ of exocarp tissue, and 1.84 mg ascorbic acid g DW^−1^ of seed. As fruit grew and developed, a decrease of ascorbic acid was observed for the mesocarp and seed but instead the exocarp displayed a mild increase of ascorbic acid during fruit growth and development. Defilippi et al. (2015) reported for Hass avocado subjected to ripening at 20°C during 15 days storage, that both tartaric acid and ascorbic acid remained relatively constant during this ripening period. The edible portion of Hass avocado has been reported to contain 8.8 mg ascorbic acid/100 g FW (Dreher and Davenport, 2013). It is important to highlight the cross talk between fruit firmness (cell wall related) and ascorbic acid content since their biosynthetic pathways share GDP-D-mannose epimerase (Mounet-Gilbert et al., 2016).

A very important group of organic acids present in avocados correspond to phenolic acids and fatty acids. Galacturonic acid, a very important component of pectins, will also be addressed in the section related to cell wall metabolism. However, Pedreschi et al. (2014) reported for Hass avocado at harvest, higher content of galacturonic acid in fast ripening fruit indicative of different physiological age. Phenolic acids do not participate in primary metabolism but mainly in secondary metabolism being precursors of other phenolic compounds and components of the cell wall. Thus, a very brief introduction to studies on the topic is incorporated in this review. Hurtado-Fernández et al. (2014, 2015a) evaluated 13 different avocado cultivars including Hass at two different ripeness stages (at physiological maturity and ready to eat ripeness stages) and reported that the concentration of organic acids and phenolic acids generally decreased as the fruit ripened, except for phenolic acids such as ferulic and *p*-coumaric which increased in concentration as the fruit ripened. Hass avocado at harvest presented a lower amount of benzoic acid compared to the ready to eat stage. But, the quinic acid concentration in Hass was higher at harvest and decreased at the ready to eat stage (Hurtado-Fernández et al., 2014). Lately, Hurtado-Fernández et al. (2016) reported the evolution of six important metabolites from the phenyl-propanoid pathway including phenolic acids (pantothenic, p-coumaric, and ferulic acid) and epicatechin, chlorogenic acid, and abscisic in different avocado cultivars over different harvest seasons depending on the variety. Results showed that the phenolic acids previously mentioned were quite similar in the four avocado cultivars studied but the concentrations of chlorogenic acid, epicatechin, and abscisic acid differed.

## Metabolism of Amino Acids During Avocado Fruit Growth, Development, and Ripening

Amino acid metabolism provides precursors for protein synthesis, for respiration processes and for a range of specialized metabolites. Fruit imports amino acids from the phloem and less from the xylem *via* symplastic or apoplastic routes depending on the stage of development (Zhang et al., 2015). Amino acids and their derivatives have been reported to exert influence on taste and quality.

A thorough study on avocado fruit amino acid metabolism during growth, development, and ripening is not available up to our knowledge. The potential physiological role of polyamines involved in nucleic and protein synthesis in avocado was studied back in 1986 by Winer and Apelbaum. These authors studied the evolution of polyamines during avocado development and ripening. The levels of putrescine and spermidine decreased during fruit growth, being these decreases moderate during fruit development until maturation. However, the most striking decrease of these polyamines was observed during ripening (Winer and Apelbaum, 1986). Coincident with the climacteric peak of ethylene, putrescine, spermidine, and spermine decreased their contents to 0 and 20% of their initial values, respectively. Exogenous application of polyamines provoked a decrease in the level of endogenous ACC in avocado fruit. Interestingly, spermine in the mesocarp remained constant while the fruit was attached to the tree. From these findings, it was postulated that the high level of polyamines in avocado fruit during development provoke the repression of ethylene production and lack of ripening while the fruit remains attached to the tree (Winer and Apelbaum, 1986).

Avocado mesocarp contains a high content of 2% protein (Dreher and Davenport, 2013). During “Hass” avocado fruit ripening, variations in the content of soluble proteins have been reported (Blakey et al., 2010, 2012). Blakey et al. (2012) reported increases of soluble protein content during ripening of Hass avocado from 17.8 mg g^−1^ DW to 34.8 mg^−1^ g DW from day 2 to day 16. This increase in soluble protein content is associated with the ripening process which demands *de novo* synthesis of enzymes/proteins involved in the different pathways triggered by the ripening process. Previously, Blakey et al. (2010) reported similar increases in soluble protein content during ripening while C_7_ sugars decreased while C_6_ sugars tended to increase as ripening proceeded.

Recent studies related to the understanding of differences in ripening speed of avocado fruit have demonstrated the participation of amino acid metabolism, protein folding, transport, and translation (Pedreschi et al., 2014; Fuentealba et al., 2017; Uarrota et al., 2019). Glutamic acid has been reported to be highly correlated to a heat shock treatment (38°C for 1 h) that triggered ripening synchronization in early and middle season Hass avocado fruit. In the same study, differences 1 day after harvest, at the metabolome and proteome levels were observed between early and middle season fruit indicating differences in the physiological state at harvest and thus different response to the effectiveness of the heat treatment in terms of ripening (Uarrota et al., 2019). Previously, in a study of Pedreschi et al. (2014) fast ripening Hass avocado fruit at harvest displayed higher contents of glutamic and aspartic acids and alanine compared to the slow ripening phenotype. These differences were attributed to differences in physiological maturity at harvest not evident with classical commercial assessment of dry matter content and firmness. Fuentealba et al. (2017) recently reported higher expression of proteins involved in correct protein folding, translation and protein *de novo* synthesis in the fast ripening Hass avocado phenotype and higher amount of aminopeptidases and carboxypeptidases involved in post-translational modification in the slow ripening Hass avocado phenotype.

## Lipid Metabolism During Avocado Fruit Growth, Development and Ripening

Avocado is a very complex matrix formed by a wide variety of compounds and one of the main components is lipids mainly composed of triacylglycerols (TAGs). TAGs composed of monounsaturated fatty acids (MUFA; 9.80 g/100 g) are the predominant ones and also of polyunsaturated (PUFA) and saturated fatty acids (SFA; 2.13 g/100 g). The abundance of these substances, together with the fact that some of the main health benefits of avocado have been attributed to its high monounsaturated fatty acid content, makes lipids one of the most studied chemical families in avocado (Hurtado-Fernández et al., 2018).

Avocado lipids can be divided into (1) neutral lipids or also called triacylglycerols (TAGs), fats or oils (tri, di, and monoacylglycerols); (2) phospholipids; and (3) glycolipids. The neutral lipid fraction constitutes 96% of the total lipid content of avocado, mainly are TAGs. TAGs composed of 18:1, 18:2, 16:0, and 16:1 fatty acids are present, and the relative concentration (percentage of total lipid) of each is in the range 59–81% (18:1), 7–14% (18:2), 7–22% (16:0), and 3–11% (16:1). It is important to point out the differences in lipid content and profile in the mesocarp, exocarp, and seed in different cultivars of avocado. For instance, Galvao et al. (2014) reported average mesocarp fat contents (g/100 g) of 16.2, 13.6 and 11.9 for Fortuna, Collinson and Barker cultivars. Average seed fat contents (g/100 g) for the same cultivars corresponded to 1.8, 2.5, and 2.1, respectively and average fat content of the peel corresponded to 0.9, 1.7, and 1.1, respectively, for Fortuna, Collinson and Barker cultivars. In terms of fatty acid composition of the mesocarp except for Barker that presented a higher % of SFA, in the other cultivars Fortuna and Collinson, MUFA were the predominant fatty acids. The seed instead of Fortuna was characterized by a higher relative content of SFA followed by PUFA and MUFA, but the other two cultivars presented a higher proportion of PUFA followed by SFA and MUFA, respectively. The exocarp instead of the three avocado cultivars was characterized by a higher relative content of MUFA followed by SFA and PUFA, respectively.

Oil content increases in the mesocarp a few weeks after the fruit sets. As oil increases in the mesocarp, water content decreases by the same amount, so that the total percentage of oil and water remains constant during fruit life. Nevertheless, biosynthesis of triglycerides does not start at the beginning of the physiological life of the fruit and can change during fruit development and ripening (Ozdemir and Topuz, 2004). Avocado fruit is characterized by the presence of lipid-containing idioblasts, specialized cells, distributed uniformly in the mesocarp, which make up to 2% volume of the edible portion and are characterized by a thick (4 μm) wall consisting of three layers of cellulose, suberin, and lignified cellulose. Idioblasts contain high acetogenin content. Acetogenin profiles are conserved in mesocarp, while seed profiles are dynamic during fruit growth (Rodríguez-López et al., 2017). During avocado fruit development, the mesocarp accumulates by dry weight 60–70% oil and 10% carbohydrates. The oil is stored in the form of triacylglycerol (TAG) and is predominantly composed of oleic acid (Kilaru et al., 2015). It has been observed that the increase in fruit weight is highly correlated with the accumulation of lipid content in the mesocarp tissue (Kilaru et al., 2015) and unlike mature oilseeds, mature avocados cv. Hass can accumulate up to 18% oil in the mesocarp. The avocado seed instead did not present much change in accumulation during growth and development of the fruit (Kilaru et al., 2015).

### Fatty Acid Biosynthesis During Avocado Fruit Growth, Development and Ripening

At least six different metabolic pathways requiring the activation of over 200 genes are involved in oil metabolism in avocado fruit (Salas et al., 2000; Kilaru et al., 2015). Thus, sucrose to be converted into TAG requires the degradation of sucrose and generation of pyruvate in the plastid requiring the active participation of glycolysis, pentose phosphate pathway and plastid transporters and the synthesis of fatty acids in the plastid and TAG assembly in the endoplasmic reticulum. It consists of three events: (1) production of the glycerol backbone; (2) formation of fatty acids or fatty acyl moieties; and (3) esterification of glycerol with the fatty acid components to yield TAGs (Bewley et al., 2013). Carbohydrates are the carbon sources in plants for TAG biosynthesis. *De novo* fatty acids synthesis takes place in the plastid, acetyl-CoA is needed as precursor and it is derived from sucrose and by the action of two key enzymes acetyl-CoA carboxylase and fatty acid synthase, palmitate, stearate, and oleate are formed as main products. These fatty acids are then exported to the cytosol where they are activated to Acyl-CoAs and further modified. The desaturases introduce double bonds, and elongases elongate the fatty acid chain length. Kilaru et al. (2015) reported for Hass avocado at five different developmental stages high expression of transcripts levels for the orthologs of ribulose 1,5 biphosphate carboxylase (RBC), phosphoribulokinase, and phosphoenolpyruvate (PEPC) in avocado mesocarp, and it was consistent with their suggested role in carbon assimilation. In addition, same authors reported high expression of sucrose synthase (SuSy) in the cytosol during mesocarp development and highlighted the major role of SuSy in the generation of the hexoses needed for pyruvate synthesis. Starch is the main substrate for glycolysis in the plastids and in the same study of Kilaru et al. (2015), transcripts for starch synthesis and degradation gene orthologs were abundant through the different avocado mesocarp developmental stages. It has been previously reported that at early stages of fruit set in avocado, about 44% of the flesh weight is due to sugars which continue to increase during rapid fruit growth but then decline during oil accumulation (Liu et al., 1999b). One fourth of total oil content accumulation in avocado has been reported to occur during early stages of development and Vergara-Pulgar et al. (2019) have recently reported enrichment of transcripts related to lipid localization, fatty acid synthase activity and acyl carrier activity involved in fatty acid biosynthesis when comparing mesocarp of 150 DAFS (days after fruit set) with 240 DAFS, respectively. Acetyl-CoA carboxylase the regulatory enzyme that controls the rate of fatty acid synthesis and catalyzes the first reaction to generate malonyl-CoA was also detected in avocado and oil palm (Dussert et al., 2013; Kilaru et al., 2015). As previously mentioned, avocados are rich in C_7_ sugars and it is postulated by Kilaru et al. (2015) that they might play in early stages of fruit growth, a regulatory role in the initiation of oil biosynthesis.

Kilaru et al. (2015) carried out a complete transcriptomic analysis targeting pathways related to oil metabolism in mesocarp and seeds from avocado cv. Hass at five different developmental stages (from 125 to 230 g FW ~ 4 to 12.5% oil content) and compared with that of oil rich monocot (palm oil) and dicots (rapeseed and castor) tissues in order to identify tissue and species specific regulation of TAG biosynthesis supposed to be considered a highly conserved process in plants. Avocado mesocarp presented about 45, 34, 9, 5, 4, and 3% (average of five developmental stages) of the transcripts to be genes involved in glycolysis and plastidial fatty acid biosynthesis, pentose phosphate pathway, sucrose degradation, phospholipid, and TAG assembly and plastid transporters, respectively (Kilaru et al., 2015). It was noted that across the five developmental stages of the mesocarp, the abundance of genes involved in glycolysis for the generation of pyruvate and further fatty acid synthesis was striking as the genes involved in the acyl group synthesis in the plastid in contrast to the abundance levels of genes involved in phospholipid and TAG assembly remained pretty constant among the different developmental stages of the mesocarp. Vergara-Pulgar et al. (2019) has recently reported a strong expression of transcripts related to fatty acid and TAG biosynthesis at the start of the developmental process of Hass avocado (150 DAFS and 240 DAFS) but downregulation for later stages of 300 DAFS and 390 DAFs, respectively, which support previous reports that most of the lipid content in avocado is produced during the first half of fruit development, then the processes are reduced but not culminated. Same authors have recently reported oleosin to be differentially expressed in the mesocarp between 240 DAFS and 390 DAFS possibly having a role as lipid storage stabilizer. Previously, Horn et al. (2013) and Gidda et al. (2013) reported two new lipid droplet associated proteins (LDAP1 and LDAP2) in avocado mesocarp. Kilaru et al. (2015) found higher expression of these proteins during the five evaluated developmental stages in avocado mesocarp. These proteins are supposed to bind and stabilize lipid particles in avocado mesocarp. Recently, Ge et al. (2019) reported a histological analysis of lipid droplets in avocado mesocarp during fruit development (from 65 days after pollination, DAP to 125 DAP for the avocado variety Guitenda N°2, *Persea americana* var. guatemalensis). Small lipid droplets of 2 μm of diameter were present throughout the periphery of the cells from 65 to 105 DAP. But at 125 DAP (harvest date corresponding to 11.12% total fatty acids based on dry mass), larger lipid droplets of 10–25 μm occupying the volume of most of the cell were observed.

Kilaru et al. (2015) indicate based on this transcriptomic data that for oil rich tissues and diverse species, a conserved common stoichiometry and temporal regulation of transcripts with oil accumulation holds. However, only some orthologs of the fatty acid biosynthetic pathway in avocado were similar to that of monocots and dicots. The orthologs expressed in avocado for PDHC-E1β (pyruvate dehydrogenase complex E1β enzyme), HAD (HAD superfamily, subfamily IIIB acid phosphatase), and FATA (acyl ACP thioesterase A) were different than the one predominantly expressed in oil rich tissues of monocots and dicots. Thus, Kilaru et al. (2015) reported more than 60% of the transcripts encoding for fatty acid biosynthesis pathway belonged to stearoyl ACP desaturases (SAD/DES) and to ACP and their expression increased with the maturity of the mesocarp. ACP transcripts represented 24% of the total fatty acids synthesis gene expression, transcripts that mapped to ACP4 (acyl carrier protein 4, plastid) were by far the most abundant in avocado while the other four isoforms were barely detectable.

The expression pattern of stearoyl-ACP desaturase genes in avocado mesocarp reflects its lipid composition according to Kilaru et al. (2015). During mesocarp development, transcript levels coding for FAB2 were the most abundant than any other enzyme involved in lipid biosynthesis and represented 44% of the total plastidial fatty acid synthesis gene expression and increased during maturation and correlated with the increased oleic acid of avocado mesocarp. This was supported by Kilaru et al. (2015), who studied five different developmental stages of Hass avocado (I–V, from 125 to 230 g fresh weight and 4.5–12.5 lipid content fresh weight) and reported 18% of the total lipids to be polyunsaturated during stages I–III and then less than 10% were polyunsaturated in stages IV and V, respectively. The lipid composition agreed with the higher expression levels of LPCAT (lysophosphatidylcholine acyltransferase) and PDAT (phospholipid:diacylglycerol acyltransferase) in stages I–III compared to IV and V. Thus, a possible role for acyl editing during early stages of development is proposed and supported by higher levels of transcripts for an ortholog of oleate desaturase (FAD2) during early stages of development (I–III) compared to IV and V respectively. Recently, Ge et al. (2019) reported the fatty acid mesocarp composition during four stages of avocado fruit development (65–125 DAP; corresponding to 9.45–54.75 g of mesocarp mass) and the expression of key genes and regulators of glycolysis and fatty acid biosynthesis. The composition of fatty acids varied at the four stages of development. The concentration of palmitic and palmitoleic acids increased from 65 to 105 DAP followed by a rapid increase until 125 DAP. Oleic acid content only increased after 85 DAP and then increased dramatically by 70 at 125 DAP. Linoleic acid content fluctuated during fruit development and was higher than those of the other fatty acids, but linolenic acid content declined constantly during fruit development. These results showed that the content of palmitic, oleic, and linoleic acids all increased during the four stages of fruit development, reaching their maxima at the late stage. In addition, the expression of fatty acid related genes such as *PaWRI1, PaACP4-1, PaACP4-2,* and *PapPK-β1* significantly increased during fruit development with maximum expression at 125 DAP. From 65 to 125 DAP, *PaWRI1, PaACP4-2,* and *PapPK-β1* displayed a fourfold increase but kept showing significant differences among the four stages of fruit development but *PaACP4-1* only increased 0.5–1.0-fold expression at 65 DAP and 1.42 fold at 125 DAP. Thus, the observed total fatty acid changes during the four stages of avocado fruit development by Ge et al. (2019) were consistent with the changes of expression of *PaWRI1, PaACP4-2,* and *PapPK-β1* but inconsistent with *PaACP4-1* and *PaWRI2* expression patterns.

Up to our knowledge, the only complete study based on the avocado transcriptome of *P. americana* var. *drymifolia* related to patterns of genes involved in fatty acid metabolism and fruit ripening corresponds to Ibarra-Laclette et al. (2015). The transcriptome of avocado fruit from three different ripening stages corresponding to pre-climateric, climacteric and post-climateric was reported. From these data and based on the expression of transcripts related to “fatty acid synthesis” and “fatty acid elongation, desaturation and export” for these three postharvest ripening stages, Ibarra-Laclette et al. (2015) suggested that the initiation of fruit ripening determines the end of oil accumulation and fatty acid composition. Most of the genes expressed in the fruit homologous to fatty acid biosynthesis genes decreased as fruit ripened. These authors reported three homologs (UN066747, UN21149 and UN33083) of FAB2/SSI2 (stearoyl-acyl carrier protein desaturase) (AT2G43710) as differentially expressed unigenes during ripening. FAB2/SSI2 corresponds to the major enzyme that converts stearic acid (C_18:0_) to monounsaturated acid (C_18:1_) in chloroplasts. These results together with those of Kilaru et al. (2015) based on fruit growth and development of avocado support that lipid accumulation and changes in the fatty acid profile such as fatty acid desaturation occur during fruit development, and possibly, this process concludes a few days after harvest (pre-climacteric stage). The evident decrease of FAB2/SSI2 transcripts and genes involved in fatty acid biosynthesis during fruit ripening explains the non or minimal changes observed in the fatty acid composition during postharvest ripening of avocado. Ibarra-Laclette et al. (2015) hypothesize ethylene as the signaling molecule once perceived that halts lipid biosynthesis and programs changes in the fatty acid profile.

Minimal change in the fatty acid composition of the mesocarp during postharvest has been reported, and the increased oil concentration during storage and ripening has been related to postharvest dehydration and to increased lipid recovery due to partial cell wall breakdown (Mostert et al., 2007; Meyer and Terry, 2008). Pedreschi et al. (2016) reported no effect of postharvest ripening strategies such as temperature and ethylene on the fatty acid profile and content of Hass avocados. Instead the fatty acid profile is determined during growth and development and highly influenced by temperature, light, and photoperiod. Low temperatures during growth and development have been reported to favor the accumulation of polyunsaturated fatty acids having oxygen as the limiting factor for desaturases and membrane fluidity limit the activity of desaturases. Several studies have reported differences in the profile of fatty acids of Hass avocado from different origin (Donetti and Terry, 2014; Pedreschi et al., 2016), which can have direct consequences on critical cold storage temperature. On this perspective, García-Rojas et al. (2012) reported potential candidate genes involved in loss of quality of late season Hass avocados stored under cold storage grown in Chile and identified stearoyl-ACP desaturase (*PamSAD*) and acyl-CoA synthase (PamACoAS1,2,3). Furthermore, Gudenschwager et al. (2013) on an attempt to understand darkening of the Hass avocado mesocarp under suboptimal cold storage, studied pattern expression of genes that encode the multi-subunit acetyl CoA carboxylase enzyme (MS-ACCase) as being key enzymes in fatty acid biosynthesis and potentially playing a role in membrane synthesis and maintenance. Two MS-ACCase subunits were identified *in silico*, a biotin carboxylase (BC) and a biotin carboxyl carrier protein (BCCP). Since these two genes (*PamACCase-BC* and *PamACCase-BCCP*) were increased in ripe avocados after cold storage, the authors suggested that these genes might be related to cold storage induced physiological disorders in Hass avocado. In addition, several studies have reported that biosynthesis of antifungal compounds in avocado fruit is connected with the activation of fatty acid biosynthetic pathways. Different elicitors (cold temperature, ethylene, and fungal infection) reported by Madi et al. (2003) and Wang et al. (2004) enhanced the expression/activity of Δ9 stearoyl-ACP desaturase (SAD) and Δ12 oleate desaturase (FAD2).

### Fatty Acid Catabolism During Avocado Fruit Development and Ripening

Fatty acids to be further utilized by most lipid metabolic enzymes need to be thioesterified by long chain acyl CoA synthases (LACS). Nine different isoforms of LACS have been identified in *Arabidopsis* and in avocado mesocarp, transcripts for the ortholog of LACS4 have been reported as most abundant followed by LACS8, LACS1, and LACS9 (Kilaru et al., 2015). More than 80% of the transcripts of LACS orthologs were represented by the ER-associated isoforms (LACS1, LACS4, and LACS8) and only 16% by the ortholog of plastidial LACS9 in avocado mesocarp. Thus, it remains to be elucidated which of the LACS contributes to acyl activation and where it happens. In addition, orthologs of peroxisomal LACS6 and LACS7 were barely detectable in avocado mesocarp during fruit development. Thus, these results suggest that fatty acids undergo little β-oxidation during mesocarp development (Kilaru et al., 2015). Fatty acids can be degraded *via* peroxisomal β-oxidation, and it is supposed to operate during lipid synthesis or incorporated into triacylglycerols *via* the Kennedy pathway in the endoplasmic reticulum (Kessel-Vigelius et al., 2013).

Kilaru et al. (2015), reported up to 34 different lipase genes during five developmental stages of which four corresponded to monoacyl glycerol lipases (MAGL) and two to triacylglycerol lipases (TAGL). Two MAGL genes did not present changes in expression during the five developmental stages, while two other MAGL tended to decrease in expression as the developmental stage advanced. For the TAGL genes, no differences in expression were observed for the five developmental stages. These results confirm reduced expression of lipases during oil accumulation and furthermore during maturation, which confirms previous studies that reported no changes in oil content and fatty acid profile during avocado ripening.

### Oil Regulation in Avocado Fruit

Previous studies of oil palm mesocarp revealed WRI1 to be correlated with oil accumulation. In avocado mesocarp, Kilaru et al. (2015) reported in addition to WRI1, transcripts for the isoforms WRI2 and WRI3 to be highly expressed but upstream regulators of WRI1 such as LEC1, LEC2, and FUS3 were not expressed or detected in avocado mesocarp. These results indicate that oil regulation in non-seed tissue is different than in seed tissues.

In avocado mesocarp, the overall expression pattern of WRI1 orthologs was similar to genes WRI1, which regulate ACP, BCCP (biotin carboxyl carrier protein of heteromeric ACCase), KASII (ketoacyl-ACP synthase II), and PDHC (pyruvate dehydrogenase) and the pattern of oil accumulation. In contrast to the role of WRI2 in *Arabidopsis* fatty acid biosynthesis, the high expression of this ortholog in avocado mesocarp during oil accumulation suggests a potential role in TAG accumulation in avocado. Ge et al. (2019) recently reported that while *PaWRI1, PaACP4-1,* and *PapPK-β1* seem to play key roles in the accumulation of oil in avocado mesocarp as also reported by Kilaru et al. (2015), the pattern of expression of *PaWRI2* differed indicating that this transcription factor contrary to what was reported by Kilaru et al. (2015) might not be influencing oil accumulation.

## Cell Wall Dynamics Metabolism During Fruit Development and Ripening in Avocado

Avocados undergo a dramatically softening of the mesocarp during its climacteric period, and these changes in mesocarp firmness are accompanied by changes in skin color, influencing the fruit quality and storability (García-Rojas et al., 2016). Fruit softening is primarily associated to a cell wall disassembly attributed to modifications induced by ripening with an impact in the primary cell wall polysaccharides (Brummell, 2006). There is a consensus in the literature on the conformation of the primary cell walls of plants, including fruits, which corresponds to a pectin matrix and a cellulose-xyloglucan network (Brummell, 2006; Caffall and Mohnen, 2009). It is well known that cell wall structure can have an impact on fruit texture (Brady, 1987; Yakushiji et al., 2001; Vicente et al., 2007; Ng et al., 2014). During the maturation of avocados highly dynamic alterations have been described, involving mainly enzymatic cell wall degradation (Jeong et al., 2002, 2003; Defilippi et al., 2018). Despite the relevant implications of the remodeling of the cell wall and the physiology of avocado, there is very little information in early stages of fruit development.

### Cell Wall Dynamics During Ripening of Avocado Fruit

Pectins are mainly composed of homogalacturonans (HG), rhamnogalacturonan type I and II (RG-I and RG-II, respectively; Ishii and Matsunaga, 2001; Coenen et al., 2007). The galacturonic acid (GalA) is the main constituent of the HG, which is secreted to the cell wall where they are de-esterified by the action of pectin methylesterase (PME) (Micheli, 2001; Baiano et al., 2011); leaving available sites for the action of polygalacturonase (endo-PG, exo-PG) and pectate lyases (PL, Brummell, 2006). During avocado softening, the cell wall content undergoes several modifications and pectins are the most studied component. The content of galacturonic acid decreases significantly throughout the softening. These changes in GalA content have been correlated with an increase in the PG activity (Jeong et al., 2002; Jeong and Huber, 2004). Moreover, during avocado softening, a polygalacturonase gene has been described showing an increased expression in postharvest storage (García-Rojas et al., 2012). Although PG participates in the softening of the fruit, this enzyme would not be so relevant in the phase when most significant loss of firmness of the fruit occurs (Jeong et al., 2003). Pectin modifications have been described as a polysaccharide solubilization, mainly in HG, and it is highly linked to PME and PG activity (Awad and Young, 1979; Wakabayashi, 2000). Consequently, these changes in the pectin matrix have been proposed as the main causes of avocado softening as a result of cell wall loosening (Jeong et al., 2002).

Additionally, several publications point out the importance of the modifications of the lateral chains of RG-I in the firmness of fruit (Gross, 1984; Orfila et al., 2001; Ng et al., 2014; Cornuault et al., 2018). RG-I consists of a backbone of rhamnose and galacturonic acid, with side chains of arabinan, galactan, and arabinogalactan, which can be linked to hemicelluloses/celluloses (Vidal et al., 2001; Guillon et al., 2017). Wang et al. (2018) indicated that RG-I content is three-fold the RG-II in the fruit cell wall, and RG-I represented the most important branched pectic component of the primary cell wall and middle lamella (Goulao et al., 2012). β-galactosidases and α-arabinosidases have been described as enzymes that remove galactan and arabinan side chains of RG-I in fruit ripening (Brummell, 2006). In avocados, the pectin distribution has not been described. However, RG-I has been recently associated with the softening in avocados, where galactose (the main component of the side chains of this polysaccharide) amount decreases as firmness decreases. Moreover, β-galactosidase activity increases during softening (Tateishi et al., 2001, 2007; Defilippi et al., 2018). Another component of the RG-I side chains, arabinose, also showed a decrease during softening and an α-L-arabinofuranosidase activity increase have been reported (Kamiyoshihara et al., 2018). Additionally, the rhamnose content remained constant suggesting that the changes observed in RG-I correspond to a disassembly of the RG-I side chains and not to its backbone structure (Sakurai and Nevins, 1997; Defilippi et al., 2018).

Hemicelluloses correspond to cell wall polymers that interact with cellulose (Hayashi and Kaida, 2011) and pectins (Fasoli et al., 2016). Hemicelluloses are conformed by linear chains of glucose, xylose, and/or mannose and possess different side chains involving these monosaccharides and fucose or arabinose. Xyloglucans are the most important polysaccharides in the fraction of hemicelluloses of the primary wall of dicots (Nishinari et al., 2007). In avocados, little is known about the hemicellulose dynamics during softening. Nevertheless, it has been reported that endoglucanase could play an essential role in xyloglucan remodeling (Hatfield and Nevins, 1986; O’Donoghue and Huber, 1992). Another cell wall enzyme, xyloglucan endotransglycosylase/hydrolase participates in the remodeling of hemicelluloses (Fasoli et al., 2016), presenting in tomato a high activity during the expansion of the fruit and either decreases or remains constant during maturation (Miedes and Lorences, 2009). However, in avocados no transcriptional or proteomic characterizations have been reported.

Expansins promote the elimination of hydrogen bonds between cellulose and hemicellulose fibers, and indirectly the degradation of pectins by increasing access to pectin remodeling enzymes (Cosgrove, 2000; Wang et al., 2018). In avocados, expansins are poorly understood, showing a little detection in ripen avocadoes (Rose et al., 2000). Likewise, cellulase activity has been widely studied in avocados. Similarly, to pectin-degrading enzymes, cellulase detection and activity increase during avocado softening, changing the elastic properties of cellulose matrix (Pesis et al., 1978; Sakurai and Nevins, 1997; Jeong et al., 2002; Jeong and Huber, 2004).

### Agricultural Practices to Mitigate Cell Wall Disassembly in Avocado Fruit

Cold storage is extensively used by the avocado producers to improve the postharvest viability by delaying ripening-associated softening of avocados. In refrigerated storage, the cell wall of avocados continues to undergo modifications such as degradation of pectins by PG, PME, and β-Gal activities and cellulose degradation by endoglucanase activity at a slower rate (Chen et al., 2017; Defilippi et al., 2018). Defilippi et al. (2018) determined that the activity of PG and β-Gal increased in avocadoes after cold storage compared to harvest, which correlates with the depolymerization of HG and the solubility of pectin polysaccharides and reduction of flesh firmness. However, although this cold storage technology is critical in influencing avocado softening delay, there is insufficient information describing the cell wall changes in pectins, hemicellulose, and cellulose that contribute to the firmness loosening in cold-stored avocados.

The use of plant growth regulators (PGR) is a common practice in some tree fruit handling. One of the most studied PGR in fruit ripening and softening is ethylene because it plays a crucial role in the development of climacteric fruits by initiating and coordinating the ripening process (Jeong and Huber, 2004). Thus, 1-methylcyclopropene (1-MCP) binds to the ethylene receptor and prevents this gas from having a physiological effect (Sisler and Serek, 1997). 1-MCP has been widely used to delay the ripening process in avocados (Feng et al., 2000; Zhang et al., 2011). Defilippi et al. (2018) described that treatments with 1-MCP can extend the postharvest life of the avocado, delaying the enzymatic activity of PG, PME, and β-Gal. Likewise, Tateishi et al. (2007) described that during treatments with 1-MCP, the β-Gal activity is delayed due to negative transcriptional regulation of these genes, impacting directly in the galactosidase proteins. Furthermore, the endoglucanase activity is also repressed during the treatments with 1-MCP, altering the composition and structure of the polysaccharides conforming the cell wall (Jeong et al., 2002; Jeong and Huber, 2004).

### Post-genomic Tools for the Understanding of Cell Wall Metabolism in Avocado

The combination of “omics” techniques such as metabolomics, proteomics, and transcriptomics would help to better understand the processes that occur in avocado fruit as it has been used in other plant species (Shiratake and Suzuki, 2016; Fabres et al., 2017; Savoi et al., 2017). The study of the proteins involved in the development of the plants and the maturation of the fruit provides valuable information to understand processes, in particular, the metabolism of the remodeling of the cell wall (Vicente et al., 2007; Palma et al., 2011; Nilo et al., 2012; Shi et al., 2014; Tenhaken, 2015; Pedreschi, 2017; Martínez-Esteso and Bru-Martínez, 2018). In the case of avocado fruit, at the proteomic level, some analyses have been carried out to understand some physiological processes, such as ripening (Fuentealba et al., 2017), or for the identification of the protein profiling of avocados (Esteve et al., 2012; Righetti et al., 2015). About transcriptomic techniques, different efforts have been carried out to the study of fatty acid synthesis (Ibarra-Laclette et al., 2015; Kilaru et al., 2015), and the responses to both biotic and abiotic stresses (Chanderbali et al., 2009; Mahomed and van den Berg, 2011; Reeksting et al., 2014, 2016). A transcriptomic analysis revealed an increase in the expression of different gene clusters associated to cell wall macromolecule catabolic processes during avocado ripening, inducing changes in the composition of the cell wall. Moreover, genes involved in cell wall biogenesis showed a decreased expression during ripening (Ibarra-Laclette et al., 2015). Additionally, a recent transcriptomic study during avocado fruit development described that genes codifying cell wall structural proteins showed a constantly decreased expression through fruit development, suggesting a role of these genes in avocado fruit firmness. These genes are homologs to proline-rich proteins (PRPs), which constitute a large part of the cell wall structural proteins (Vergara-Pulgar et al., 2019). Furthermore, certain metabolomic studies have been developed in the understanding of the biology and evolution of this fruit (Contreras-Gutiérrez et al., 2013; Hurtado-Fernández et al., 2015b, 2016), the description of processes from the secondary metabolism (Rodríguez-López et al., 2015; Meléndez-González and Espinosa-García, 2018), and the ripening process of avocado (Pedreschi et al., 2014, 2016). However, there is a lack of information regarding the interactions between avocado cell wall remodeling, fruit development and postharvest modifications and changes regarding variety, agronomical cultural practices, and postharvest technology. Alternatively, these changes may impact on the cell wall metabolism that later translates into avocado firmness evolution. Therefore, by having physiological data and omics information (i.e., transcriptomics, proteomics, and metabolomics), we will be able to correlate these data with the firmness properties of the fruit, which will provide more precise ideas about the changes in the processes studied and their impact on the quality of avocados.

## Conclusions and Perspectives

A deep understanding of primary metabolism of avocado fruit during growth, development, and ripening and its relation to fruit quality is still to be achieved. Even though, the most recent studies have approached the understanding of this fruit species using new sequencing platforms and bioinformatic tools to critically study and deal with a non-model organism that lack a reference genome, still there is need for further studies that include a complete profiling of carbohydrates, lipids and hormones together with transcriptomic changes in mesocarp, peel, and seed. Further studies that incorporate stable isotope labeling approaches might be important to predict the flux of carbon in central metabolism in avocado. Thus, in depth understanding of avocado fruit development and carbon partitioning could be achieved. Amino acid and organic metabolism have been practically ignored in the studies reported. The role of C_7_ sugars in early stages of mesocarp development and regulation of fruit ripening remains to be elucidated. In addition, the potential role of C_7_ sugars and its coordination with the initiation of lipid biosynthesis remains to be studied. Regulation of oil biosynthesis in avocado mesocarp still deserves further studies, since fruit quality is determined in this fruit basically by the total oil content and composition of fatty acids. Cell wall metabolism has been poorly studied up to date in avocado and is crucial since it completely influences fruit firmness and other texture and sensory-related properties.

## Author Contributions

RP, CF, and JEA significantly contributed to the sections related to sugar, organic acid, and amino acid metabolism. In addition, they designed the outline of the content of the manuscript and leaded the editing of the manuscript. CM, VU, and RP significantly contributed to the section related to oil metabolism. RC-V, PO, and BD significantly contributed to the section related to cell wall metabolism.

### Conflict of Interest Statement

The authors declare that the research was conducted in the absence of any commercial or financial relationships that could be construed as a potential conflict of interest.
